# Assessment of the impact on compliance of a new CPAP system in obstructive sleep apnea

**DOI:** 10.1007/s11325-012-0651-0

**Published:** 2012-01-28

**Authors:** Alison J. Wimms, Glenn N. Richards, Adam V. Benjafield

**Affiliations:** ResMed Science Centre, ResMed Ltd, 1 Elizabeth Macarthur Drive, Bella Vista 2153, Sydney, Australia

**Keywords:** Obstructive sleep apnea, Continuous positive airway pressure, Treatment compliance, Adherence

## Abstract

**Background:**

Despite the efficacy of continuous positive airway pressure (CPAP) for the treatment of obstructive sleep apnea (OSA), compliance with therapy remains suboptimal. The aim of this study was to determine whether the use of S9^TM^ increased compliance in established CPAP users.

**Methods:**

Subjects with OSA (50) were recruited into the study. When subjects entered the study, 28 days of respective compliance data were downloaded from the patient's usual CPAP device. Subjects trialled the S9 CPAP for 28 days. Subjects then resumed use of their usual CPAP for 28 days. Compliance data from the patient's usual CPAP pre- and post-trialling S9 were compared with data from the S9 CPAP.

**Results:**

Patients were significantly more compliant when using the S9 than their usual CPAP device both pre- and post-S9 based on average daily usage. CPAP pre-S9 = 6.58 ± 1.95 (mean hours ± SD), S9 = 7.08 ± 1.18 h and CPAP post-S9 = 6.71 ± 1.72 h. The difference between CPAP pre-S9 and S9 was 0.5 h (*p* = 0.003). The difference between S9 and CPAP post-S9 was 0.35 h (*p* = 0.01). There was no significant difference between CPAP pre-S9 and CPAP post-S9 (*p* = 0.34). Patients also completed questionnaires comparing the S9 system to their usual device. Subjective feedback showed a strong preference for the S9.

**Conclusions:**

Participants were significantly more compliant when using the S9 than their usual CPAP device both pre- and post-S9 use.

## Introduction

Obstructive sleep apnea (OSA) is a serious condition associated with several adverse consequences, including increased incidence of cardiovascular morbidity and mortality [1, 2]. Continuous positive airway pressure (CPAP) is known as the gold standard treatment for OSA and has been shown to reduce the risk of cardiovascular fatal and non-fatal events and decrease mortality rates compared with untreated OSA [3–5]. In adherent patients, CPAP therapy has also been shown to improve the symptoms associated with OSA, such as excessive daytime sleepiness and reduced quality of life [6].

Despite its efficacy, compliance with CPAP is often suboptimal. When compliance is defined as at least 4 h usage per night, between 29% and 83% of patients are considered non-compliant [7]. To address this compliance issue, modifications have been undertaken to CPAP devices in order to improve comfort and acceptance by patients.

The main technological advances to CPAP since its invention have been heated humidification, auto-adjusting CPAP and pressure reduction on exhalation. Many studies have attempted to review the effect of these technological advances on compliance with little success. A recent meta-analysis reviewed 3 studies of 135 subjects, which examined compliance differences between heated humidification and no humidification. The results were mixed, with two parallel group trials finding no significant difference in compliance and one cross-over study finding a small but significant improvement in usage in the humidification group (5.7 h) compared to placebo humidification (5.3 h) [8]. Recently, heated tubing has been developed to compliment CPAP humidification. A randomised cross-over study undertaken to investigate whether heated tubing would improve compliance in 44 patients found no improvement in compliance with heated tubing compared with standard CPAP [9].

A meta-analysis was also conducted on studies comparing auto-adjusting CPAP (APAP) and fixed pressure CPAP. Thirty studies with a total of 1,136 subjects were reviewed. The analysis found no significant difference in usage in parallel group trials and a small statistically significant difference in usage of 0.21 h (13 min) in favour of APAP from cross-over studies [8]. A similar meta-analysis conducted 1 year later identified 19 studies of 845 patients. This analysis found a mild improvement (0.23 h) in compliance with APAP compared with CPAP [10].

Pressure reduction on exhalation has also been investigated for its potential effect on compliance. A meta-analysis of six studies comparing pressure reduction on exhalation to fixed CPAP with a total of 318 participants found no significant difference in adherence to therapy [8]. This outcome was replicated by a recent randomised controlled trial of 184 subjects which found that patients using reduced pressure on exhalation had comparable usage to those using standard CPAP, although they were trending towards increased usage [11].

The latest design of a CPAP device, known as S9^TM^ (ResMed Ltd, Bella Vista, Australia), encompasses new features, including a humidification system with heated tube, enhanced APAP algorithm, improved motor technology and reduced noise. The investigators hypothesised that the combination of these new features may lead to an increase in CPAP compliance. The aim of this study was to examine whether compliance with the S9 device would be greater than the patient's usual CPAP system. The secondary aim was to assess the usability of the S9 compared with the patient's usual CPAP device.

## Materials and methods

### Patient selection

Fifty patients with OSA, established on CPAP therapy (≥ 6 months), were recruited into this study. Patients were recruited from the ResMed Sleep Trials Registry, a voluntary registry open to all OSA patients using CPAP. All patients provided written informed consent, and the study was approved by the University of NSW Human Research Ethics Committee.

Inclusion criteria were age >18 years; established on CPAP (fixed pressure or APAP) therapy for at least 6 months; usual use of a ResMed mask, humidifier and CPAP device with ability to download data; and willingness to provide written informed consent. Patients who were using bi-level therapy were excluded. Table 1 displays subject demographics.

**Table 1:** Subject demographics

	CPAP pre-S9/S9	CPAP post-S9
Number of subjects	50	44
Male/female (%)	64/36	61/39
Mask—pillows/nasal/full face (%)	40/36/24	43/36/21
Device—S7/S8/S8 II (%)	16/22/62	14/18/68
Mode—AutoSet/CPAP (%)	64/36	61/39
Humidifier—yes/no (%)	98/2	98/2

### Assessment period

A period of 28 days was selected as the assessment time for each stage. Short term usage tends to predict longer term compliance, and it is common for studies investigating device changes to measure compliance after 4 weeks [9, 12].

### Baseline CPAP assessment

At the baseline assessment, a retrospective download of the last 28 days was taken from the patient's usual CPAP device (CPAP pre-S9). The download included usage hours, AHI, pressure and leak. At this time, subjective feedback on the patient's usual device was collected through an 11 point Likert scale questionnaire. The questionnaire addressed comfort of breathing on the device, dryness of the nose and mouth, amount of condensation in the tube and mask, noise of the device and feeling of being refreshed each morning.

### Device settings

All subjects were provided with an S9 system which included an H5i heated humidifier and heated tube (ClimateLine). The subject's prescribed therapy settings and usual mask remained the same throughout the trial. Patients were provided written and oral instructions on how to use the S9 system before returning home. Patients were also given access to a helpline in case of any problems.

### S9 assessment 

After trialling the S9 for 28 days, a download of the S9, including usage hours, AHI, pressure and leak, was performed. Subjective feedback regarding the S9 was collected using the same questionnaire as described in the baseline assessment. Subjects also selected which CPAP system they preferred (their usual CPAP, the S9 or no preference).

### CPAP post-S9 assessment

**Fig. 1:**
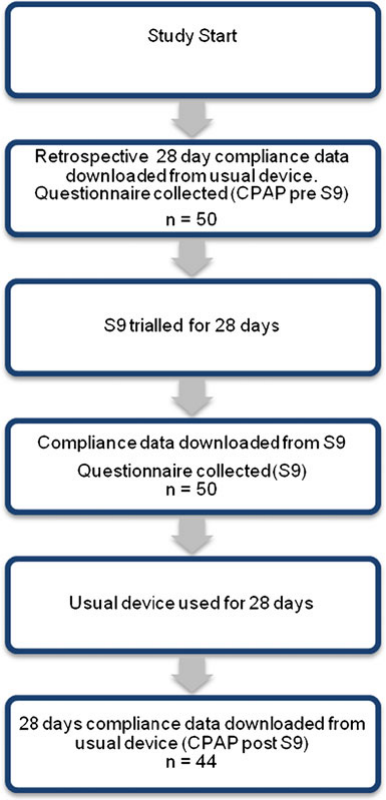
Flow chart

After completing the trial of S9, subjects resumed use of their usual CPAP system. During this period, participants were again offered access to a help line and received the same amount of clinical care received during the S9 assessment. After 28 days, they either attended the Sleep Research Centre for a download of their device or sent in the data card from their device. The protocol flow chart is shown in Fig. 1.

### Analysis

Compliance data, leak and pressure downloaded from the subject's usual CPAP device and the S9 device were analysed using the Paired *T*-Test for normal distributions and the Mann–Whitney test and chi-square test for non-normal distributions. Subjective data from the usability questionnaires were analysed using the Wilcoxon Signed Ranks test. Unless stated, data are presented as mean ± standard deviation. Statistical tests were considered significant when *p* ≤ 0.05. Analysis was undertaken with Statistica (version 8; OK, USA) and MiniTab (version 16, PA, USA) data analysis software. A priori power calculation undertaken on an unpublished pilot study indicated that a sample size of 47 was required to give 80% power to detect a 0.5-h change in average daily usage.

## Results

### Study sample

All subjects completed the CPAP pre-S9 baseline visit and S9 assessment, and 44 completed the CPAP post-S9 assessment. Of the six subjects who did not complete the CPAP post-S9 assessment, one had the device stolen, one subject became involved in another clinical trial, one subject purchased an S9 to replace their usual device, and three patients did not respond to investigator follow up.

### Compliance

Average daily usage on the S9 system increased 30 min from a mean of 6.58 ± 1.95 h on the patient's usual CPAP before trialling S9 to 7.08 ± 1.18 h when trialling S9 (*p* = 0.003). The S9 was also used an average of 21 min longer than the subject's usual device when they resumed use post-trialling S9, with the mean average usage on the S9 of 7.07 ± 1.2 h compared with the subject's usual device usage post-S9 of 6.72 ± 1.72 h (*p* = 0.010). Compliance on the subject's usual device was not significantly different pre- and post-trialling the S9, with the average daily usage of 6.57 ± 1.98 h before trialling the S9 compared to 6.72 ± 1.72 h after trialling the S9 (*p* = 0.34). Table 2 displays the outcomes of the data downloaded from the devices.

**Table 2:** Compliance data

	Number	CPAP pre-S9	S9	CPAP post-S9	*P*
Average daily usage (hours)	50	6.58 ± 1.95	7.08 ± 1.18		0.003^a^
	44		7.07 ± 1.2	6.72 ± 1.72	0.010^a^
	44	6.57 ± 1.98		6.72 ± 1.72	ns

^a^Values considered significant at <0.05

**Fig. 2:**
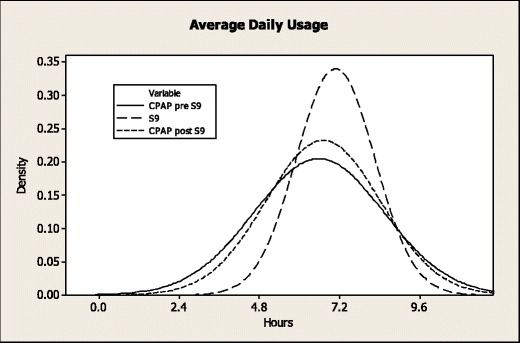
Distribution of average daily usage

Figure 2 shows reduced variation in the distribution of average usage of S9 compared with the subjects' usual CPAP with the lower levels of usage absent during the S9 phase. The variance of average daily usage was significantly different (*p* = 0.001) on the S9 (IQR 1.346) compared with the subject's usual device pre-S9 (IQR 1.883). There was also a significant difference (*p* = 0.011) in variance of S9 compared with CPAP post-S9 (IQR 1.692). There was no difference in variance of CPAP pre-S9 vs. CPAP post-S9 (*p* = 0.40)

There was a significant difference between average days used < 4 h on the usual CPAP pre-S9, compared with S9 days used < 4 h. The usual CPAP pre-S9 was used < 4 h an average of 2.04 days, whereas the S9 was used < 4 h an average of 1.00 day (*p* = 0.003). There was no significant difference between days used < 4 h on the S9 (1 day) and average number of days used < 4 h on the patient's usual device post-CPAP (1.64 days) (*p* = 0.134) There was also no significant difference between days used < 4 h on the usual CPAP pre-S9 (1.54 days) and days used < 4 h on the usual CPAP post-S9 (1.50 days) (*p* = 0.277).

**Fig. 3:**
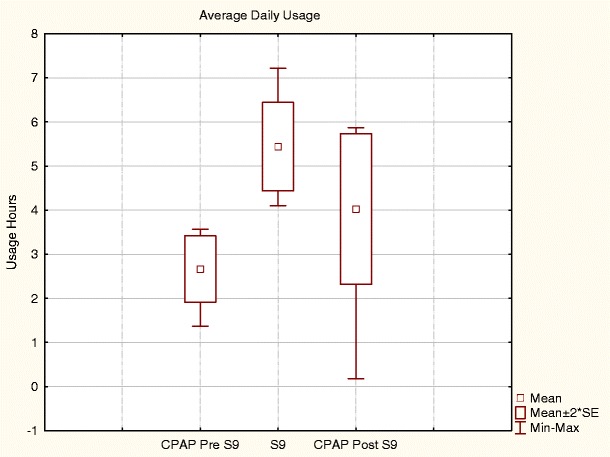
Average daily usage of participants with < 4 h average daily usage pre-S9. *p-value < 0.05

Six subjects had an average daily usage of less than 4 h on their usual device before trialling the S9 (Fig. 3). These subjects improved their average daily usage from 2.67 ± 0.9 h on their usual CPAP pre-S9 to 5.43 ± 1.23 h on the S9 (*p* = 0.001). There was a decrease in usage hours when these patients resumed their usual CPAP post-S9 from 5.43 ± 1.23 h on S9 to 4.03 ± 2.07 h on their CPAP post-S9 (*p* = 0.041). There was no significant difference in average daily usage between CPAP pre-S9 and post-S9 (*p* = 0.080). Mean daily usage pre-S9 of the drop out group (*n* = 6) was not significantly different from the other participants with average daily usage of 6.75 ± 1.87 h during CPAP pre-S9, compared with 6.57 ± 1.98 h for the rest of the participants (*p* = 0.83). S9 average daily usage of this drop out group was also not significantly different to the rest of the participants, with 7.22 ± 1.05 h compared with 7.07 ± 1.2 h for the rest of the participants (*p* = 0.76). When the drop out group is excluded from analysis, the 44 remaining subjects had a significant (*p* = 0.005) 30 min greater average daily usage on S9 compared to baseline (6.57 ± 1.98 vs. 7.07 ± 1.2 h)

There were no significant clinical differences in any of the other therapy parameters, including leak, pressure and AHI downloaded from the devices, as shown in Table 3.

**Table 3:** Data downloaded from CPAP devices

	CPAP pre-S9 (*n* = 50)	S9 (*n* = 50)	CPAP post-S9 (*n* = 44)
95%ile mask leak (L/min)^a^	17.82 ± 11.50	16.39 ± 11.04	15.91 ± 10.28
95%ile pressure (cm H_2_O)^a^	11.25 ± 1.66	11.40 ± 2.00	11.11 ± 1.63
Apnea hypopnea index^b^	4.90 ± 3.14	1.54 ± 1.60	4.47 ± 3.29

^a^In comparisons across all groups *p* = ns

^b^The S9 uses a revised AHI scoring method; therefore, direct comparison of AHI between S9 and patient's usual device is not valid

### S9 usability

**Fig. 4:**
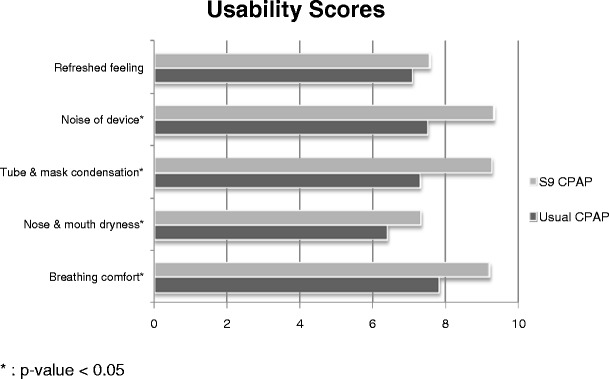
Average usability scores of the S9 and the patient's usual CPAP

Subjects rated the ease of use of the S9 an average of 9.48 ± 1.03 out of 10, which is significantly higher than the set criterion score of 6 (*p* < 0.001). Subjects rated the S9 significantly better than their usual device in comfort of breathing (*p* < 0.001), dryness of nose and mouth (*p* = 0.027), amount of condensation in the tube and mask (*p* < 0.001) and noise (*p* < 0.001). No difference was found in the feeling of being refreshed after using each device (*p* = 0.066). Figure 4 displays the average usability scores for the S9 and the patient's usual CPAP. In terms of overall preference, 78% preferred the S9 system, 16% preferred their usual device, and 6% found no difference between the devices.

## Discussion

The primary result of the study was that compliance improved significantly with the use of the S9 system. Average daily usage on the S9 system was improved by 30 min compared to the subject's CPAP pre-S9, and compliance was still significantly improved on the S9 compared with the subject's usual CPAP device post-trialling S9. Subjective feedback showed a strong preference for the S9. This study is one of only a small group to show a significant increase in compliance based on device changes.

The adverse effects of untreated OSA are well documented and have been shown to be reversible with CPAP therapy. Without treatment, the patient continues to be exposed to the characteristic pauses in breathing and oxygen desaturations synonymous with OSA. Therefore any increase in CPAP compliance is considered clinically relevant.

Several studies have examined the effect of technology changes on compliance with little success. In general, studies comparing heated humidification to no humidification have not been able to show that humidification alone increases compliance [8]. A study in 1999 by Massie et al. examined whether the introduction of heated humidification, to alleviate CPAP induced nasal congestion, would improve compliance. They found that compliance with CPAP was significantly improved by 35 min when heated humidification was added [13]. Even with the use of heated humidification, patients may still experience nasal irritation and dryness or excessive condensation in the mask and tube, which may explain why heated humidification alone is not usually able to increase compliance.

Another common complaint of CPAP users is that it can be uncomfortable or difficult to breathe on higher pressures. The development of APAP (auto-adjusting positive airway pressure) devices which vary the pressure delivered during the night have aimed to overcome high pressure discomfort. APAP devices have the advantage that they are able to keep the mean CPAP pressure lower and only increase the pressure as needed to maintain airway patency. A study examining compliance in patients using fixed pressure CPAP compared with APAP found a small increase of 12 min in the mean use on APAP (4.2 h/night) compared with fixed pressure CPAP (4.0 h/night) [14]. A similar study by Massie et al. found that the average nightly use was increased in APAP mode versus CPAP mode (5 h 6 min vs. 4 h 31 min) in patients who require pressures above 10 cm H_2_O. The study found that patients reported more restful, better quality sleep due to less discomfort from pressure [15]. Nolan et al. found that APAP and fixed pressure CPAP were both efficacious, and there was no significant difference in compliance on each device. They did find that patients requiring a high fixed pressure (≥ 8 cm H_2_0) preferred APAP mode, while those requiring a lower fixed pressure (< 8 cm H_2_0) generally preferred the fixed pressure mode [16]. While some studies have shown a preference for APAP over CPAP, a meta-analysis of 30 studies was unable to find any significant differences in parallel group trials [8]. This may indicate that APAP mode alone only increases compliance in certain patient groups.

CPAP users may complain of difficulty exhaling against positive pressure, and some CPAP devices decrease pressure delivered at the initiation of exhalation, followed by an increase in the pressure at the onset of inhalation. This technology has been examined in a number of trials reporting compliance [11, 12, 17–20]. These studies have suggested that the lowering of expiratory pressure does not substantially improve CPAP compliance, with only one study showing higher usage (4.8 h per night) compared with CPAP only (3.5 h per night) [17].

Another complaint of patients using CPAP therapy is the noise of the device, with one study finding that this was the main complaint about CPAP equipment [21]. No studies have examined the effect of a reduction in noise on CPAP compliance.

This study examines the effect of a combination of multiple technology improvements. S9 has a new humidification system with heated tube. This allows a greater output of constant humidification with minimal condensation despite varying temperature changes. Modifications have also been made to the APAP algorithm to enhance user comfort when breathing on the device, and the S9 system is also up to 78% quieter than ResMed S8^TM^ II in conducted noise. From this study, it is not possible to separate the effect of each technological improvement on compliance. However, as studies which have only examined one technological enhancement have generally been unsuccessful at increasing compliance, it is proposed that it is the combination of the improvements in humidification, enhanced APAP algorithm, and reduced motor noise that has lead to the increase in compliance seen in this study.

It may be particularly difficult to increase compliance in this group as they are already accustomed to CPAP and have settled into a routine, which adds weight to the value of the increased compliance. During the study, these established users were set up on the same treatment settings and mask system that they were using prior to enrolment in the study. This suggests that the changes to compliance were due to the technology improvements in the S9. The limitations of this study were the following: There was no randomisation or blinding of devices, and there was potential influence from receiving clinical care. This study attempted to overcome the lack of no randomisation and blinding by measuring the objective outcome of usage, and subjects were not informed of this objective. The investigators attempted to overcome any clinical care influence by using a sequential ABA protocol, where subjects continued to receive the same amount of clinical care, and access to a helpline, when they resumed use on their own PAP device. Subjects were all volunteers who had joined a registry of CPAP patients interested in taking part in research. Although this may suggest that these subjects could be biased towards new technologies, similar studies run by the researchers have found that subjects of clinical trials to test potential new products are often very critical of products which are not yet commercially available, as they have very high expectations and a personal desire to have improved PAP products. It is possible that the effect of being in a study and receiving clinical care may have lead to the non-significant 9-min increase in compliance from the patient's usual device pre-S9 when they were not enrolled in the study to post-S9 when they were in the study receiving clinical care; however, this does not account for the significant increase in usage of 21 min from the S9 to the patient's usual device post-S9, as patients were receiving clinical care during both of these study phases. In future studies, a randomised, blinded protocol should be used in order to remove any potential influence of clinical care.

In poorly compliant patients (*n* = 6), usage was improved at an average of 2.77 h from 2.67 h on their CPAP pre-S9 to 5.43 h on S9, which is considered compliant (> 4 h per night). These subjects then significantly decreased usage by 1.4 h when returning to their own device post-S9. However, when comparing usage on the subject's usual device pre- and post-S9, these subject's usage increased by 1.37 h when they returned to their usual device post-trialling S9. This mean increase is higher than the 21-min increase seen in the entire group. In this group of poorly compliant subjects, being in a study and receiving clinical care may have a larger effect on compliance than subjects who are already considered compliant. It is also possible that these poorly compliant patients noticed an improvement in OSA related symptoms when their usage increased during the S9 trial, which encouraged them to increase their nightly CPAP use when they returned to their usual device. However, as their compliance was still significantly higher on the S9 device than their usual CPAP both pre- and post-S9, it appears that the device has a large influence on compliance regardless of clinical care or increased motivation.

In conclusion, this study has shown that it is possible to improve compliance based on technology changes alone. Given the potential cost of untreated OSA to healthcare systems, it is important that adherence to CPAP therapy continues to be improved.
